# Fatty Liver Index (FLI) is the best score to predict MASLD with 50% lower cut-off value in women than in men

**DOI:** 10.1186/s13293-024-00617-z

**Published:** 2024-05-17

**Authors:** Lucilla Crudele, Carlo De Matteis, Fabio Novielli, Ersilia Di Buduo, Stefano Petruzzelli, Alessia De Giorgi, Gianfranco Antonica, Elsa Berardi, Antonio Moschetta

**Affiliations:** 1 Department of Interdisciplinary Medicine, University of Bari “Aldo Moro”, Piazza Giulio Cesare N. 11, 70124 Bari, Italy; 2 INBB National Institute for Biostructure and Biosystems, Viale Delle Medaglie d’Oro 305, 00136 Rome, Italy

**Keywords:** MASLD, Liver steatosis, Gender difference, Non-invasive tests, Metabolism, Gut-liver axis

## Abstract

**Background:**

Metabolic dysfunction-associated steatotic liver disease (MASLD) is defined by the presence of hepatic steatosis, detected on ultrasonography (US) imaging or histology, and at least one of criteria for Metabolic Syndrome diagnosis. Simple non-invasive tests (NITs) have been proposed as an acceptable alternative when US and biopsy are not available or feasible but have not been validated for MASLD. In this observational study, we investigated the reliability of NITs for MASLD detection and whether sex-differences in screening methods should be considered.

**Methods:**

We included 1069 individuals (48% males and 52% females) who underwent their first clinical examination for Metabolic Syndrome in the period between January 2015 and December 2022. Liver steatosis was detected through US and anthropometric and clinical parameters were recorded.

**Results:**

Liver steatosis was detected in 648 patients and MASLD was diagnosed in 630 subjects (355 males; 275 females). Women with MASLD showed better metabolic profile and lower prevalence of Metabolic Syndrome criteria than men. Among NITs, Fatty Liver Index (FLI) showed the best ability for detection of MASLD, with a cut-off value of 44 (AUC = 0.82). When considering the two sexes for MASLD detection via FLI, despite no substantial differences regarding FLI correlations with metabolic biomarkers except for age, women showed marked lower FLI cut-off value (32; AUC = 0.80) than men (60; AUC = 0.80).

**Conclusions:**

In this study, we found that FLI is the best non-invasive predictor of both liver steatosis and MASLD. The finding that in women FLI cut-off value for MASLD detection is 50% lower than in men suggests the need of a sex-specific personalized program of screening and prevention of dysmetabolism-related liver diseases, despite outwardly healthy biomarkers profile.

**Supplementary Information:**

The online version contains supplementary material available at 10.1186/s13293-024-00617-z.

## Abstract (plain language summary)

Fatty liver disease is caused by the accumulation of fat into the liver and it is associated to increased risk of chronic diseases. Diagnosis of fatty liver is based on biopsy or ultrasound assessment but when these procedures are not available or feasible also some non-invasive scores have been showed to be reliable measures of this condition. In this study we compared the use of ultrasound and non-invasive scores to assess liver steatosis and associated metabolic disease, finding that Fatty Liver Index (FLI) is the best score for these diagnosis. Surprisingly, in women FLI cut-off value is 50% lower than in men, suggesting that different sex-specific factors may come into play in the development and evolution of liver steatosis. Thus, we suggest the need of a sex-specific personalized program of screening and prevention of dysmetabolism-related liver diseases.

**Supplementary Information:**

The online version contains supplementary material available at 10.1186/s13293-024-00617-z.

## Highlights

- Simple non-invasive tests (NITs) have been proposed to assess liver steatosis and fibrosis but have not been validated for MASLD, a disease that shows different features and prevalence in the two sexes;
- We show here that Fatty liver Index (FLI) is the best NIT for predicting MASLD and that its cut-off value is 50% lower in women than in men;
- We suggest the need of a sex-specific personalized program of screening and prevention of dysmetabolism-related liver diseases, despite outwardly healthy biomarkers profile.

**Supplementary Information:**

The online version contains supplementary material available at 10.1186/s13293-024-00617-z.

## Background

Metabolic dysfunction-associated steatotic liver disease (MASLD) is characterized by the presence of hepatic steatosis, detected on imaging or histology, and at least one of criteria for Metabolic Syndrome diagnosis [1]. This definition of MASLD patients is quite recent, since the old nomenclature of Non-alcoholic Fatty Liver Disease (NAFLD) has been replaced only in the last years, with the dual aim of underscoring the role of the term “steatotic liver disease” (SLD) as a comprehensive term encompassing all causes of liver steatosis and the huge impact of dysmetabolic conditions in the pathogenesis of fatty liver disease and related complications [1]. It is estimated that MASLD affects more than 30% of the adult population worldwide, with its prevalence increasing on a year-to-year basis [2]. Indeed, several factors such as obesity, poor nutrition, dyslipidaemia, and sedentary lifestyle leading to lipotoxicity, reactive oxygen species production, dysbiosis of intestinal microbiome, and the induction of proinflammatory immune mediators, have been proposed as mechanisms associated with MASLD independently or in conjunction with genetic factors [3].

Impaired fasting glycemia, diabetes and prediabetes, metabolic syndrome, dyslipidaemia and adiposopathy are characterized by dysmetabolism, the metabolic derangement in glucose and lipid pathways. Such diseases may be present alone or in combination, but they all share a role in increasing the risk for cardiovascular diseases, atherosclerosis, fatty liver, and even cancer. For instance, the loss of canonical correlations among metabolic biomarkers and MASLD markers was recently found in a cohort of patients years in advance they received the diagnosis of colorectal cancer, thus suggesting that any perturbation of metabolic pathways could lead to carcinogenesis [4]. From a molecular point of view, a paradigm of such derangement is represented by nuclear Liver X Receptor (LXR) whose activation, in physiological conditions, is triggered by high intracellular cholesterol thus enhancing cholesterol efflux and catabolism. Paradoxically, in cancer cells as well as during liver regeneration, LXR activity is abated in spite of increased intracellular cholesterol levels [5]. Furthermore, adiposopathy (i.e. the dysfunction of enlarged adipocytes due to excessive fat infarction), diabetes, atherosclerosis are strictly linked to systemic low grade chronic inflammation, another factor that accelerates the progression from MASLD to steatohepatitis. Also, alterations in gut microbiota composition should be considered in the context of dysmetabolism: diabetes-associated dysbiosis affects gut wall integrity, leading to endotoxemia, chronic inflammation, and insulin resistance. Thus, chronic inflammation and insulin resistance self-feed, worsening dysbiosis and accelerating diabetes clinical progression and the onset of related diseases [6].

Regarding sex differences, the prevalence and severity of MASLD are higher in men than in women during the reproductive age, while after menopause MASLD occurs at a higher rate in women, suggesting that estrogens are protective [7]. Furthermore, estradiol regulates fatty acid synthase expression in liver and adipocytes and saturated fatty acids increase endoplasmic reticular stress and free radical production in mitochondria, which promotes the cellular damage and liver steatosis [8].

A prompt diagnosis is mandatory to prevent complications such as type 2 diabetes [9] and cardiovascular diseases (CVD) [10] and eventually reverse MASLD, also in the light of poor pharmacological options clinicians have to their bow. Ultrasonography (US) is the first-line tool for the diagnosis of hepatic steatosis in clinical practice, but represents a relatively subjective, operator-dependent, detecting technology. On the other hand, liver biopsy may not be considered for screening or follow-up, considering its invasiveness, rare but severe complications, sampling variability, and the need for short-term repeated evaluation in MASLD patients [11, 12]. In this context, major efforts to develop simple, non-invasive tools that can be used in routine clinical settings have been done and a number of serum-based non-invasive tests (NITs) have been proposed as an acceptable alternative [13], even when US is not available or feasible [14]. However, transitioning from NAFLD to MASLD definition, diagnostic criteria shifted from the exclusion of secondary causes to the detection of those dysmetabolic features that occur in patients with adiposopathy, and NITs role in MASLD has yet to be clarified. Among these NITs, according to expert consensus statement on MASLD, only the Fatty Liver Index (FLI) is considered appropriate, given the available data on its diagnostic and prognostic performance [15].

In this observational study with a cohort of 1069 out-patients suspected of fatty liver disease, we investigated if NITs can be used as non-invasive biomarkers for hepatic steatosis detection and whether sex-differences in screening methods should be considered, aiming to pave the way for early detection and personalized strategies of prevention for MASLD in both women and men.

## Methods

### Study design and patient involvement

We included 1069 individuals (48% men and 52% women) who underwent their first clinical examination for Metabolic Syndrome (MetS) at Internal Medicine Division “C. Frugoni” of University Hospital of Bari, Italy in the period between January 2015 and December 2022. Each patient received a unique ID and was enlisted in the electronic health register of Metabolic Diseases of the Department of Interdisciplinary Medicine at “Aldo Moro” University of Bari. The study was approved by the Ethics Committee (n.311, MSC/PBMC/2015) of the Azienda Ospedaliero-Universitaria Policlinico di Bari (Bari, Italy) in accordance with the requirements of the Declaration of Helsinki. Written informed consent for the use of clinical data was obtained from all participants in the study. In accordance with the approved Ethics Committee, only patients who were already 18 years old or more were included.

### Clinical assessment

Detailed information on reproductive history, smoking and alcohol drinking history, exposure to environmental toxics, medical history, educational level and other socioeconomic variables were recorded from each patient. Information on drug use, occupation and family history of cancer were also collected. Patients were also asked to answer specific questions about dietary and lifestyle behaviours through Chrono Med Diet Score (CMDS). CMDS is a questionnaire containing eleven food categories, including chronobiology of dietary habits and physical activity that we previously validated for assessing adherence to Mediterranean Diet and lifestyle. Lower CMDS scores indicates poorer adherence [16]. All questionnaires were administered in a row with standard operating procedures by trained personnel.

Moreover, physical examination, anthropometric measures, biochemical assessment, and abdomen ultrasound were performed. Average systolic and diastolic blood pressure (BP) were derived for each patient from three different measurements using manual sphygmomanometer. Hypertension was identified as systolic arterial blood pressure (SAP) ≥ 130 mmHg, diastolic arterial blood pressure (DAP) ≥ 85 mmHg and/or treatment with antihypertensive agents. Anthropometric assessment was performed using standardized procedures. Briefly, waist circumference (WC) was measured at the midpoint between the inferior part of the 12th costa and the anterior–superior iliac crest. Body Mass Index (BMI) was computed as weight (Kg) divided by the height squared (sqm) and subjects were characterized as overweight for BMI values between 25 and 29.9 and as obese for values above. Morning blood samples were obtained after 12 h of fasting from the antecubital veins, then biochemical markers of glucose and lipid metabolism were measured in patients’ serum. After blood clotting and centrifugation, serum was processed for analysis. Also, liver and thyroid markers were measured following standardized biochemical procedures. All biochemical measurements were centralized and performed in the ISO 9001 certified laboratories of the University Hospital of Bari. We also calculated a mix of non-invasive tests (NITs) according to published formulas (Supplementary Table 1), to assess liver steatosis or fibrosis and better represent the clinical continuum of MASLD, often progressing in steatohepatitis if undetected or without clinical intervention.

Metabolic Syndrome was diagnosed according to International Diabetes Federation (IDF) definition [17] and visceral obesity was defined for WC values above 80 cm in women and 94 cm in men. Type 2 Diabetes was diagnosed according to international criteria: HbA1c (percentage of glycosylated haemoglobin) ≥ 6.5% and/or fasting plasma glucose (FPG) ≥ 126 mg/dl and/or ongoing treatment for diabetes [18]. Carotid Artery Ultrasound was performed to assess Carotid Intima Media Thickness (IMT) and atherosclerosis was diagnosed for IMT > 0.9 mm according to current guidelines [19].

After an overnight fasting, patients underwent an abdominal ultrasound scanning performed by two expert physicians with more than 10 years of experience in ultrasonography with a 3.5–5 MHz convex probe (Esaote My Lab 70 Gold ultrasound system). B-mode ultrasound was used for assessment of fatty liver. Grade 1 (mild) is represented by a mild diffuse increase in fine echoes in the hepatic parenchyma with normal visualisation of the diaphragm and intrahepatic vessel borders. Grade 2 (moderate) is represented by a moderate diffuse increase in fine echoes with slightly impaired visualisation of the intrahepatic vessels and diaphragm. Grade 3 (severe) is represented by a marked increase in fine echoes with poor or no visualisation of the intrahepatic vessel borders, diaphragm and posterior portion of the right lobe of the liver [20]. MASLD diagnosis was based on the presence of liver steatosis identified by ultrasound and at least one of the five criteria for MetS, also considering BMI ≥ 25 kg/sqm to assess overweight or obesity alternatively to increased WC (Supplementary Table 2) [1].

### Data analysis

Descriptive statistical analyses of the study sample were performed, and results were expressed as mean ± standard deviation (SD) for numerical data, in counts and percentages for categorical data. Comparisons of continuous clinical variables between two groups were conducted with Mann–Whitney test, while chi-square test was used for comparison of proportions. p-values (p) lower than 0.05 were considered statistically significant.

Cut-off point analysis was used to determine the optimal value of NITs to detect liver steatosis and MASLD. In particular, the crucial point was defined by the largest distance from the diagonal line of the receiver operating characteristic (ROC) curve. Empirical ROC curves were plotted along with calculation of the Area under the Curve (AUC) with 95% confidence intervals (CI) and two-sided upper p-values for null hypothesis AUC = 0.5. Youden’s Index (YI), or equivalently, the highest Sensitivity + Specificity, was used to determine the optimal cut-off of each score. Correlations among continuous variables were analysed and estimated using Pearson’s correlations (r).

All analyses were performed using the NCSS 12 Statistical Software, version 12.0.2018 (NCSS, LLC Company, Kaysville, UT, USA) and GraphPad Prism, version 10 (GraphPad Software; San Diego, CA, USA).

## Results

### Baseline characteristics of study population

Our population study was homogeneous for sex (516 men and 553 women). Mean age was 57.9 ± 14.7 years. According to BMI, 37% of subjects had normal weight, 36% were overweight and 27% obese, while a clear prevalence of visceral obesity (78%) was found when considering WC. Indeed, mean WC value (99 ± 14.8 cm) was above the established cut-off for MetS diagnosis and MetS was diagnosed in 696 patients (42%), type 2 diabetes was diagnosed in 397 (43%), while mean HbA1c value of 41.9 ± 11.6 mmol/mol depicted a condition of prediabetes, and atherosclerosis was detected in 596 (57%) individuals.

Considering MASLD diagnosis, US detected liver steatosis in 648 patients (61%) and specifically mild steatosis in 342 (53% out of all cases), moderate steatosis in 199 (31%) and severe steatosis in 107 (16%). This led to diagnose MASLD in 630 patients (59%). Table 1 summarises all baseline characteristics of the population.

**Table 1:** Study population characterization (N = 1069)

Clinical variable	
Sex (M;F)	516 (48%); 553(52%)
Age (Years)	57.9 ± 14.7
BMI (Kg/Sqm)	27.4 ± 5.7
Waist Circumference (cm)	98.3 ± 15.5
FPG (mg/dL)	99.6 ± 27.5
HbA1c (mmol/mol)	41.9 ± 11.6
AST (U/L)	24 ± 10.7
ALT (U/L)	30.5 ± 18.1
GGT (U/L)	33.5 ± 33.1
Total cholesterol (mg/dL)	181.9 ± 40
HDL cholesterol (mg/dL)	54.9 ± 15.7
LDL cholesterol (mg/dL)	105.6 ± 34.8
Triglycerides (mg/dL)	118 ± 68.5
Overweight (BMI ≥ 25 and < 30)	381 (36%)
Obesity (BMI ≥ 30)	292 (27%)
Visceral obesity	829 (78%)
Metabolic syndrome	422 (39%)
Type 2 diabetes	397 (37%)
Liver steatosis us diagnosis	648 (61%)
Mild	342 (53%)
Moderate	199 (31%)
Severe	107 (16%)
MASLD	630 (59%)

Data are reported as mean ± SD (standard deviation) for quantitative variables and in percentage for categorical variables. Visceral obesity was diagnosed for Waist Circumference values ≥ 80 cm in females and ≥ 94 cm in males. Metabolic Syndrome was diagnosed when subjects had increased waist circumference plus at least two other criteria among Hyperglycaemia, low HDL, hypertriglyceridemia, and hypertension. Type 2 Diabetes was diagnosed for FPG > 126 mg/dl or HbA1c > 6.4% or ongoing anti-diabetic treatment. Mild steatosis is represented by a mild diffuse increase in fine echoes in the hepatic parenchyma with normal visualisation of the diaphragm and intrahepatic vessel borders. Moderate steatosis is represented by a moderate diffuse increase in fine echoes with slightly impaired visualisation of the intrahepatic vessels and diaphragm. Severe steatosis is represented by a marked increase in fine echoes with poor or no visualisation of the intrahepatic vessel borders, diaphragm, and posterior portion of the right lobe of the liver. MASLD diagnosis was based on the presence of liver steatosis and at least one of the five criteria for Metabolic Syndrome, also considering BMI ≥ 25 kg/sqm to assess overweight or obesity alternatively to increased WC

*BMI* Body Mass Index, *FPG* Fasting Plasma Glucose, *HbA1c* glycosylated hemoglobin, *AST* aspartate transaminase, *ALT* alanine transaminase, *GGT* Gamma-glutamyl transferase, *US* ultrasound, *MASLD* Metabolic dysfunction-associated steatotic liver disease

### Comparisons between men and women with MASLD

**Fig. 1:**
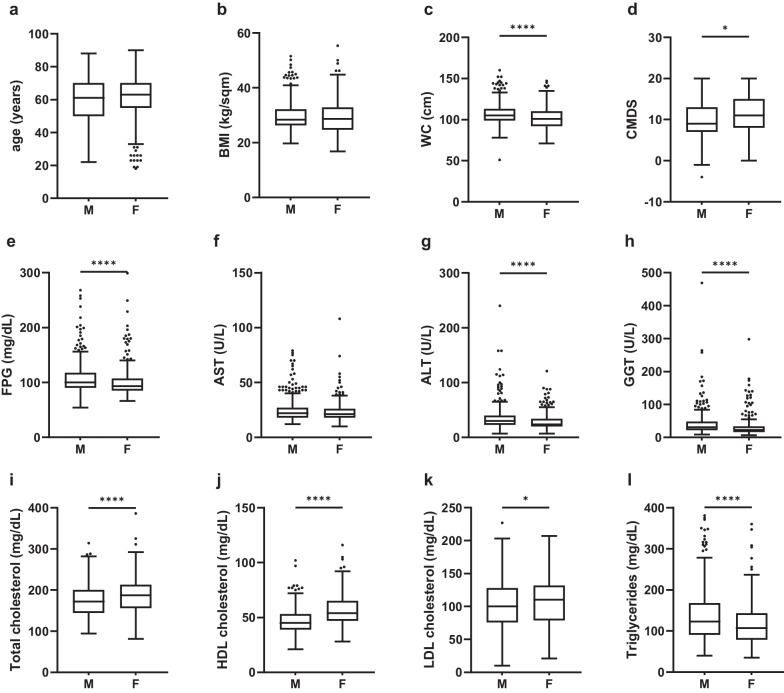
Metabolic biomarkers comparisons between males and females with MASLD. The box plots show the median (second quartile), first and third quartile, whiskers go 1.5 times the interquartile distance or to the highest or lowest point, whichever is shorter. Any data beyond these whiskers are shown as points. CMDS is a questionnaire containing eleven food categories, including chronobiology of dietary habits and physical activity that we previously validated for assessing adherence to Mediterranean Diet and lifestyle. Lower CMDS scores indicates poorer adherence. Comparisons were performed by Mann–Whitney test. Statistical significance was assessed for p-values (p) < 0.05; *p < 0.05; ****p < 0.0001. *M* males, *F* females, *BMI* Body Mass Index, *WC* Waist Circumference, *FPG* Fasting Plasma Glucose, *CMDS* Chrono Med Diet Score, *AST* aspartate transaminase, *ALT* alanine transaminase, *GGT* Gamma-glutamyl transferase

Since liver steatosis could be considered a sexual-dimorphic disease [21], with the aim of reveal any anthropometric, clinical, or metabolic biomarker that could differentiate women from men with MASLD, we then performed comparisons between MASLD male (n = 355) and female (n = 275) patients (Fig. 1).

No significant differences were detected regarding age, BMI, and HbA1c between two groups, while WC was significantly (p < 0.0001) lower in women (102.1 ± 14.5) compared to men (107.1 ± 13.6).

Considering dietary behaviours, CMDS was found significantly higher in women (10.7 ± 4.6) than men (9.5 ± 4.3), suggesting women’s better adherence to Mediterranean diet.

When comparing bio-humoral variables, female patients showed significantly lower values than men for FPG (p < 0.0001), AST (p < 0.05), ALT (p < 0.0001), GGT (p < 0.005), and triglycerides (p < 0.0001). Conversely, total (p < 0.0001), HDL (p < 0.0001), and LDL cholesterol (p < 0.05) levels were all significantly higher in women.

Since MASLD diagnosis is based on at least one criterion of metabolic impairment in addiction to liver steatosis, we compared the proportion of men and women with positive criteria for impaired fasting glycemia, dyslipidaemia, and hypertriglyceridemia as well as with hypertension, diabetes and increased adiposity assessed with both BMI and WC (Table 2). We found that both type 2 diabetes and hypertension prevalence were significantly higher in men (p < 0.005). With regard to other cardiovascular risk factors, men showed significantly higher prevalence of impaired fasting glycemia and triglyceridemia (p < 0.0001), while no difference was found for HDL-related criterion. When considering the fraction of women with increased WC, this was significantly higher than in men (p < 0.0001), while an inverse association was found for BMI (p < 0.0001).

**Table 2:** Comparisons between men and women with MASLD (N = 630)

	Men (N = 355)	Women (N = 275)	p-value
Type 2 diabetes	204 (57%)	123 (45%)	< 0.005
Hypertension criterion	213 (60%)	130 (47%)	< 0.005
Hyperglycaemia criterion	245 (69%)	142 (52%)	< 0.0001
HDL cholesterol criterion	105 (30%)	94 (34%)	ns
Triglycerides criterion	123 (35%)	54 (20%)	< 0.0001
Waist circumference criterion	309 (87%)	263 (95%)	< 0.0001
BMI ≥ 25 kg/sqm	306 (86%)	204 (74%)	< 0.0001

Chi-square test was used for comparisons of proportions. p-value < 0.05 was considered significant. Type 2 Diabetes was diagnosed for FPG > 126 mg/dl or HbA1c > 6.4% or ongoing anti-diabetic treatment. Hypertension was identified as systolic arterial blood pressure (SAP) ≥ 130 mmHg, diastolic arterial blood pressure (DAP) ≥ 85 mmHg and/or treatment with antihypertensive agents. Hyperglycaemia was diagnosed for FPG ≥ 100 and/or HbA1c ≥ 5.7% and/or ongoing anti-diabetic treatment. Waist circumference was considered pathological above 80 cm in women and 94 cm in men. To characterize dyslipidaemia, HDL cut-off was < 40 mg/dL in males and < 50 mg/dL in females, while a value of Triglycerides ≥ 150 mg/dL for both genders was considered pathological. Metabolic Syndrome was diagnosed when subjects had increased waist circumference plus at least two other criteria among Hyperglycaemia, low HDL, hypertriglyceridemia, and hypertension

*BMI* Body Mass Index, *ns* not-significant

### NITs comparison to predict liver steatosis and MASLD

**Fig. 2:**
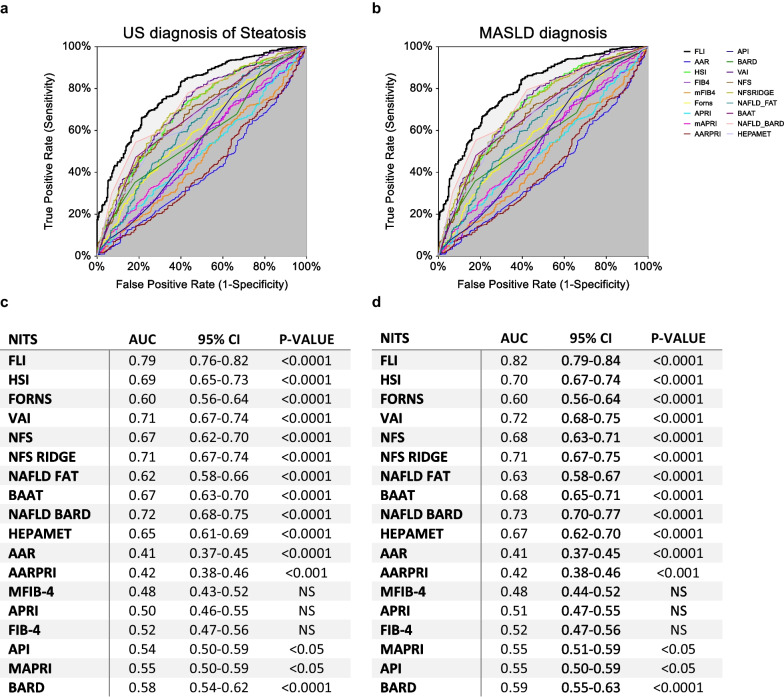
Comparison of empirical ROC curves of non-invasive tests (NITs) in prediction of liver steatosis and MASLD. ROC curves of NITs for prediction of liver steatosis assessed by ultrasound (**a**) and MASLD (**b**). The tables (**c**, **d**) show empirical estimation of area under curve (AUC) with 95% CI (confidence interval) and p-value for each NITs. Statistical significance was assessed for p-values (p) < 0.05. *US* ultrasound, *FLI* Fatty Liver Index, *HIS* Hepatic Steatosis Index, *VAI* Visceral Adiposity Index, *NFS* NAFLD Fibrosis Score, *NAFLD-FAT* NAFLD-Liver Fat Score, *BAAT* BMI-ALT-Age and Triglycerides, *AAR* AST to ALT Ratio, *AARPRI* (AST to ALT ratio) to Platelet Ratio Index, *mFIB-4* modified FIB-4, *APRI* AST to Platelet Ratio Index, *FIB-4* Fibrosis-4 index, *API* Atherosclerosis Plasma Index, *mAPRI* modified APRI, *NS* not-significant

To determine if non-invasive methods could be used in place of ultrasound, we compared ROC curves of main NITs for liver steatosis (Fig. 2a). Among significant scores, FLI showed the highest AUC (0.79, 95% CI 0.76–0.82) to discriminate liver steatosis (p < 0.0001) with a cut-off value of 55 (YI = 0.48, sensitivity 0.66, specificity 0.79).

Since MASLD is considered a travel companion of MetS and adiposopathy, and liver steatosis is necessary for its diagnosis, we then compared NITs ROC curves for prediction of MASLD (Fig. 2b). Interestingly, FLI again showed the highest AUC (0.82, 95% CI 0.79–0.84) and YI (0.49, sensitivity 0.74, specificity 0.75) with a cut-off value of 44.

### FLI correlations with main metabolic parameters in two sexes

To verify the reliability of FLI in MASLD detection for both sexes, we studied different correlations among FLI and main metabolic biomarkers in two sexes. Specifically, since FLI formula encompasses triglycerides, BMI, GGT, and waist circumference values [22], we studied FLI correlations with clinical and metabolic biomarkers not considered for FLI calculation.

**Fig. 3:**
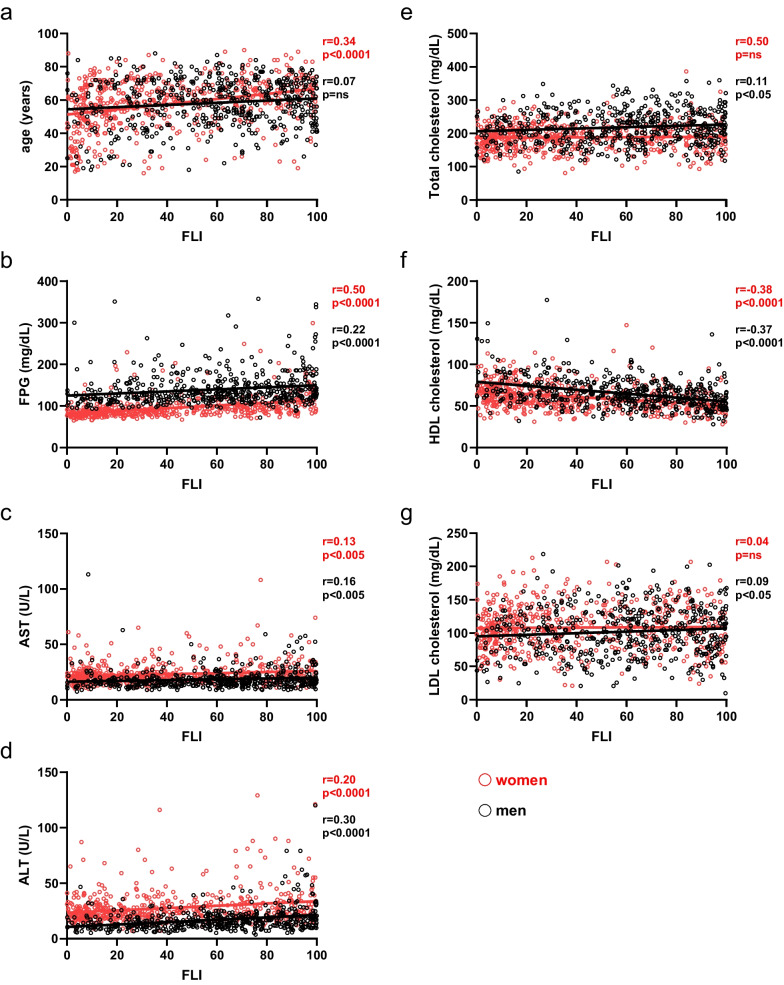
Correlations between FLI and metabolic biomarkers in men and women. FLI Pearson’s correlations (r) and p-values (p) with age (**a**), FPG (**b**), AST (**c**), ALT (**d**), Total Cholesterol (**e**), HDL Cholesterol (**f**), LDL Cholesterol (**g**) in two sexes are reported. *BMI* Body Mass Index, *FPG* Fasting Plasma Glucose, *AST* aspartate transaminase, *ALT* alanine transaminase; ns, not-significant

Age was directly correlated with FLI values only in women (r = 0.34, p < 0.0001), suggesting that age had an impact only in females’ susceptibility for developing MASLD (Fig. 3a). On the contrary, FPG (Fig. 3b), AST, and ALT (Fig. 3c, d) were directly correlated with FLI in both sexes. With regard to the lipid profile, HDL-c showed a strong inverse correlation with FLI in both sexes (Fig. 3f), while total and LDL-cholesterol were directly correlated with FLI only in men (Fig. 3e, g).

### NITs comparison for predicting MASLD in men and women

**Fig. 4:**
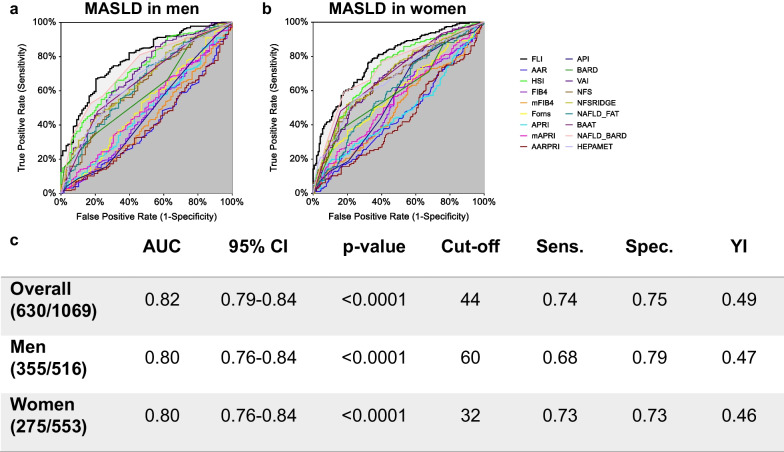
Comparisons of non-invasive tests (NITs) ROC curves for MASLD detection in men and women. ROC curves of NITs for detection of steatosis in men (**a**) and women (**b**). The table (**c**) shows empirical estimation of area under curve (AUC) with 95% CI (confidence interval) and p-value, cut-off values with related sensitivity, specificity, and Youden’s Index (YI)

Furthermore, to analyse if sex could be a considerable factor in the accuracy of NITs to identify MASLD, we performed ROC analyses of NITs in two sexes (Fig. 4a, b), confirming that FLI was the best to predict MASLD in both sexes. In men, cut-off value was 60 with a sensitivity of 0.74 and a specificity of 0.75 (AUC = 0.80; 95% CI 0.76–0.84).

In women, FLI predicted MASLD with a lower cut-off value (32) that had a sensitivity of 0.73 and a specificity of 0.73 (AUC = 0.80; 95% CI 0.76–0.84).

## Discussion

In this study, we investigated whether NITs could predict ultrasonographic diagnosis of hepatic steatosis, in a cohort of 1,069 individuals suspected of metabolic diseases, finding that, compared to a large series of validated liver steatosis scores, FLI better predicts not only liver steatosis but also MASLD.

The FLI is an algorithm proposed by Bedogni et al. [22] as a predictor of hepatic steatosis in the general population. It has been validated fatty liver diagnosed qualitatively by ultrasound with a cut-off of ≥ 60 to consider the likely presence of steatosis and < 30 to rule out its presence, with values ranging from 0 to 100. In the management of NAFLD, FLI is an important algorithm in the diagnosis and prognosis of patients with metabolic risk [23]. In previous studies, FLI was found superior to other indexes for NAFLD diagnosis [24] and to predict fatty liver by abdominal US compared to other non-invasive markers in both genders [25].

To the best of our knowledge, few studies have compared FLI to other NITs in the context of MASLD. In our cohort, we found that FLI is useful not only for steatosis detection, but also for MASLD diagnosis, with different cut-off values in the two sexes. Since liver steatosis is defined as hepatocytes infarction with triglycerides for more than 5% of liver parenchyma, the greater ability of FLI respect to other NITs may lie on its formula considering serum triglycerides levels that better reflects the lipodystrophy associated with such condition. Furthermore, also the concomitant use of both WC and BMI in FLI formula may explain why this score is able to detect not only steatosis but also the associated metabolic dysfunction. Although significantly applied worldwide, the original cut-off point of FLI proposed by Bedogni et al*.* without any stratified restrictions has already been challenged because it is difficult to apply in practice [26] and high variability for cut-off values of FLI is present in literature, since in a Kenyan cohort FLI cut-off value was actually 6.12 [27] while in western China the best cut-off value for NAFLD diagnosis was 30.42 [24], and in 12,794 Uyghur adults, the optimal cut-off values for diagnosing MASLD was 45 in both sexes [28].

An intriguing aspect of our study is the finding that FLI cut-off values are very different in two sexes. When considering US diagnosis of fatty liver, gender-based optimal cut-off of FLI has been identified as lower in women compared to men. Similarly to our findings, in a cohort of 1976 Asian subjects, women showed a FLI cut-off value of 10.927 and men a value of 34.522 for NAFLD prediction and this gender-difference was maintained also when sub-grouping for light and moderate drinking habits [29]. Similarly, in two different American cohorts, hepatic steatosis was predicted for greater FLI values in men (48.57 and 61.47) than in women (41.93 and 51.65) also when stratifying for WC and BMI [11]. This was the case in several other studies [30], which also saw a decrease in sensitivity (28.4% for men and 11.5% for women) when the cut-off point of FLI was set at 60 as in the original study [25]. On the contrary, in an Iranian cohort, FLI cut-off value for fatty liver was lower in men than women (46.9 and 53.8, respectively) [31]. In our cohort, no substantial differences between sexes were found when considering FLI relationships with other metabolic biomarkers, so we posit that FLI is a reliable index of MASLD in both genders. Moreover, the presence of a dysmetabolic state might affect women more than men with regard to liver outcomes [32], as already showed by the stronger association in women than in men between diabetes, hypertension and CVD with FLI-defined NAFLD in different studies [32–34]. Study comparisons between men and women with MASLD showed that females developed liver steatosis and MASLD even in the context of better glycaemic and lipidic profiles while the prevalence of type 2-diabetes and hypertension were higher in males than females. In line with these findings, it has been previously showed that, although diabetes prevalence is higher in males [35], in women with diabetes the risk of cardiovascular events is higher [36]. A possible explanation for our apparently paradoxical finding may lie in the fact that MASLD female patients of our cohort were mainly in post-menopausal status, thus probably they were at increased risk of hepatic steatosis since they had lost the estrogens protection. Indeed, estrogen deficiency in post-menopausal women promotes MASLD and can exacerbate histological features of MASLD while in men physiological levels of androgens protect against fatty liver disease, preventing or attenuating the consequences of obesity, insulin resistance, and other features of metabolic syndrome [37]. This may suggest us that MASLD women also have an increased risk for progression toward cirrhosis, so further follow-up studies are needed for assessing FLI prognostic role, beyond its ability to discriminate MASLD.

Thus, our results highlight the need for revised FLI cut-offs, especially in women aged 50 or more that are affected by MASLD, also in the view of direct correlation between FLI and age we found only in female subjects. Indeed, even if values of FLI seem below the original cut-off point in such individuals, the metabolic alterations that are part of the MASLD spectrum should impose to revise cut-offs according to sex.

From a biological perspective, hormonal differences in two sexes could expand on this hypothesis. With regard to FLI, testosterone, dihydrotestosterone, progesterone and 17a-hydroxyprogesterone were inversely associated with FLI in men, whereas in women a positive association of free testosterone with FLI was observed [33]. Thus, since in our cohort women had a mean age of 60 years, suggesting that they were already experimenting low-estrogen status due to their menopausal state, this difference in FLI cut-off values between sexes may be explained by the loss of estrogen protection in post-menopausal women.

From a molecular perspective, hepatic immune cell function is reshaped during MASLD and contributes to disease pathogenesis [38] and natural killer T-cells may have a different role in males and females for MASLD development since they have been showed to protect against diet‑induced steatohepatitis in a gender-specific manner [39]. Moreover, estrogen directly regulates Formyl peptide receptor 2 (FPR2) expression, which is related to estrogen-mediated protection against NAFLD [40]. Nevertheless, a crucial role of LXR in MASLD patients has been proposed since its expression was positively correlated with not only the amount of intrahepatic fat, but also with intrahepatic inflammation and hepatic fibrosis [41]. Intriguingly, activation of LXRα in transgenic mice confers a female-specific resistance to lithocholic acid–induced hepatotoxicity [42]. Moreover, LXR involvement has been proposed to explain metabolic-based gender differences in regulation of high cholesterol saturated fat diet and consequent steatohepatitis [43]. Finally, lipid metabolites derived from cholesterol, such as 27-hydroxycholesterol that activates LXR, are endogenous selective estrogen receptor modulator in the vasculature [44], thus resulting as contributing factors in the loss of estrogen protection from vascular disease, an inhibiting effect eventually translatable to the hepatic district.

Finally, also gender-difference in dietary habits should be considered. Indeed, an inverse association between FLI and adherence to Mediterranean Diet was found in a cross-sectional analysis of data from two population-based adult cohorts in England and Switzerland [45], confirming the huge impact of dietary and lifestyle factors in MASLD pathogenesis and the putative role of nutritional strategies as a complementary therapeutical approach. Our findings about women’s better adherence to Mediterranean Diet is consistent with result from a MASLD German cohort about higher mean daily salt intake in men than in women [46], but also the role of different gut microbiota composition should be investigated. Indeed, when focusing on the so called gut-liver axis, western diet-induced steatosis, insulin sensitivity, and predicted microbiota functions showed a gender-difference [47], maybe driven by the releasing of the enterokine fibroblast growth factor-15/19 (FGF-15/19) that being secreted from the gut, signals to the liver to regulate bile acids (BA) synthesis and lipid/glucose metabolism in an age-specific manner due to sex-divergent expression of BA transporters and BA synthetic enzymes [7].

### Perspectives and significance

Taken together, results of this study highlight the significance of FLI as an easy-to-use tool for MASLD detection and personalized strategies, in both sexes. Our evidence also emphasizes the need for revised FLI cut-offs in women, as this could better reflect the association between FLI‑defined MASLD and the concurrent dysmetabolic state that might affect women more than men with regard to general and liver-specific outcomes. Crucially, since most MASLD risk factors are modifiable, the use of FLI could help to implement sex- and age-specific lifestyle interventions, mainly when cardiometabolic imbalances are still subclinical, to prevent the onset of MASLD and its progression. Also prospective studies for assessing the prognostic role of NITs in predicting sex-specific evolution of liver steatosis toward fibrosis and eventually cirrhosis may be useful and welcome.

## Conclusions

In conclusion, the finding that FLI cut-off values for MASLD detection are lower in women suggests the requirement of a sex-specific personalized program of screening and prevention of dysmetabolism-related liver diseases, despite an outwardly healthy biomarkers profile.

### Supplementary Information

Supplementary Material 1.

Supplementary Material 2.

Supplementary Material 3.

## Data Availability

The data that support the findings of this study are not openly available due to reasons of sensitivity and are available from the corresponding author upon reasonable request. Data are located in the electronic health register of Metabolic Diseases of the Department of Interdisciplinary Medicine at “Aldo Moro” University of Bari.
