# Supplementary material for: Fatty Liver Index (FLI) is the best score to predict MASLD with 50% lower cut-off value in women than in men
Source: Biol Sex Differ. 2024 May 17;15:43. doi: 10.1186/s13293-024-00617-z (PMC11100212; doi:10.1186/s13293-024-00617-z)
Supplement: Supplementary file 1 — Supplementary Material 1. [file 13293_2024_617_MOESM1_ESM.docx]

**Supplementary table 1. Non-invasive tests (NITs) with their associated formulas**

| **Scores** | **Formulas** |
| --- | --- |
| FLI (1) | ${e^{y}}/{\left( 1+e^{y} \right)\times100}$Where y = 0.953 × ln(triglycerides) + 0.139 × BMI, + 0.718 × ln (GGT) + 0.053 × waist circumference – 15.745 |
| AAR (2) | $\mathrm{AST}/\mathrm{ALT}$ |
| FIB-4 (3) | $\frac{Age\times AST}{Platelet Count \times\sqrt{\mathrm{ALT}}}$ |
| mFIB-4 (4) | $\frac{10 \times Age\times AST}{Platelet Count \times ALT}$ |
| FORNS (5) | $7.811 - 3.131 \times ln\left( platelet count \right) + 0.781 ln\left( GGT \right) +3.467 \times ln(age) - 0.014 (total cholesterol)$ |
| APRI (6) | $\frac{\mathrm{AST}/{ULN of normal value} \times100}{Platelet Count}$ |
| m-APRI (7) | [Age*(AST/40)/(Albumin*Platelet Count)]*100 |
| AARPRI (8) | $\frac{\mathrm{AST}/\mathrm{ALT}\times150}{Platelet Count}$ |
| API (9) | Sum of  Age: < 30 = 0, 30-39 = 1, 40-49 = 2, 50-59 = 3, 60-69 = 4, ≥ 70 = 5  PLT: ≥ 225 = 0, 200-224 = 1, 175-199 = 2, 150-174 = 3, 125-149 = 4, < 125 = 5 |
| BARD (10) | BMI ≥ 28: No = 0, Yes = 1; AST/ALT ratio: ≥0.8 No = 0, Yes = 2; and Diabetes: No = 0, Yes = 1 |
| VAI (11) | Waist circumference/[39.68+(1.88*BMI)]*(Triglycerides/1.03)*(1.31/HDL cholesterol) |
| NFS (12) | -1.675 + (0.037$\times$age) + (0.094$\times$BMI) + (1.13$\times$IFG or diabetes [yes = 1, no = 0]) + (0.99$\times$AAR) – (0.013$\times$platelet count) – (0.66 $\times$albumin) |
| NFS-RIDGE (13) | -0.614+0.007$\times$ALT- 0.214$\times$HDL-0.053$\times$Triglycerides +0.144$\times$HbA1+0.032$\times$WBC+0.132$\times$hypertension |
| NAFLD-FAT (14) | 1.18 × metabolic syndrome +0.45× diabetes (2, if yes; 0, if no) +0.15× FSI +0.04× AST −0.94× AAR −2.89 |
| BAAT (15) | BMI≥28, age≥50, ALT≥2xULN, triglycerides≥1.7 mmol |
| NAFLD-BARD-LFS (16) | [‑2.89 + 1.18 × metabolic syndrome (Yes = 1/No = 0) + 0.45 × Type 2 diabetes mellitus (Yes = 2/No = 0) + 0.15 × fasting serum insulin (mU/L) + 0.04 × fasting AST (U/L) – 0.94 × AST/ALT] |
| HEPAMET (17) | 1/ (1+e[5.390−0.986 x Age [45-64 ]−1.719 x Age [≥65]+0.875 if Males−0.896 x AST [35-69IU/L]−2.126 x AST [≥70 IU/L]−0.027 x Albumin [4-4.49g/dL]−0.897 x Albumin [<4g/dL]− 0.899 x HOMA [2-3.99 if no DM]−1.497 x HOMA [≥4 if no DM]−2.184 x DM−0.882 x Platelet Count x 1.000/mL[155-219]−2.233 x Platelet Count x 1.000/mL[<155]) |
| HSI (18) | 8 × ALT/AST + BMI (+2, if type 2 diabetes; +2, if female) |

**Abbreviations:** FLI, Fatty Liver Index; AAR, AST to ALT Ratio; FIB-4, Fibrosis-4 index; mFIB-4, modified FIB-4; APRI, AST to Platelet Ratio Index; mAPRI, modified APRI; AARPRI, (AST to ALT ratio) to Platelet Ratio Index; API, Atherosclerosis Plasma Index; VAI, Visceral Adiposity Index; NFS, NAFLD Fibrosis Score; NAFLD-FAT: NAFLD- Liver Fat Score; BAAT: BMI, ALT, Age and Triglycerides; HSI, Hepatic Steatotic Index.

**References**

1. Bedogni G, Bellentani S, Miglioli L, Masutti F, Passalacqua M, Castiglione A, et al. The Fatty Liver Index: a simple and accurate predictor of hepatic steatosis in the general population. BMC Gastroenterol. 2006 Nov 2;6:33.

2. Botros M, Sikaris KA. The de ritis ratio: the test of time. Clin Biochem Rev. 2013 Nov;34(3):117–30.

3. Sterling RK, Lissen E, Clumeck N, Sola R, Correa MC, Montaner J, et al. Development of a simple noninvasive index to predict significant fibrosis in patients with HIV/HCV coinfection. Hepatol Baltim Md. 2006 Jun;43(6):1317–25.

4. Wang HW, Peng CY, Lai HC, Su WP, Lin CH, Chuang PH, et al. New noninvasive index for predicting liver fibrosis in Asian patients with chronic viral hepatitis. Sci Rep. 2017 Jun 12;7(1):3259.

5. Wai CT, Greenson JK, Fontana RJ, Kalbfleisch JD, Marrero JA, Conjeevaram HS, et al. A simple noninvasive index can predict both significant fibrosis and cirrhosis in patients with chronic hepatitis C. Hepatol Baltim Md. 2003 Aug;38(2):518–26.

6. Lin ZH, Xin YN, Dong QJ, Wang Q, Jiang XJ, Zhan SH, et al. Performance of the aspartate aminotransferase-to-platelet ratio index for the staging of hepatitis C-related fibrosis: an updated meta-analysis. Hepatol Baltim Md. 2011 Mar;53(3):726–36.

7. Huang C, Seah JJ, Tan CK, et al. Modified AST to platelet ratio index improves APRI and better predicts advanced fibrosis and liver cirrhosis in patients with non-alcoholic fatty liver disease. Clin Res Hepatol Gastroenterol. 2021;45(4):101528. doi:10.1016/j.clinre.2020.08.006

8. Tseng PL, Wang JH, Hung CH, Tung HD, Chen TM, Huang WS, et al. Comparisons of noninvasive indices based on daily practice parameters for predicting liver cirrhosis in chronic hepatitis B and hepatitis C patients in hospital and community populations. Kaohsiung J Med Sci. 2013 Jul;29(7):385–95.

9. Poynard T, Bedossa P. Age and platelet count: a simple index for predicting the presence of histological lesions in patients with antibodies to hepatitis C virus. METAVIR and CLINIVIR Cooperative Study Groups. J Viral Hepat. 1997;4(3):199-208. doi:10.1046/j.1365-2893.1997.00141.x

10. Harrison SA, Oliver D, Arnold HL, Gogia S, Neuschwander-Tetri BA. Development and validation of a simple NAFLD clinical scoring system for identifying patients without advanced disease. Gut. 2008 Apr 29;57(10):1441–7.

11. Amato MC, Giordano C, Galia M, et al. Visceral Adiposity Index: a reliable indicator of visceral fat function associated with cardiometabolic risk. Diabetes Care. 2010;33(4):920-922. doi:10.2337/dc09-1825

12. Angulo P, Hui JM, Marchesini G, Bugianesi E, George J, Farrell GC, et al. The NAFLD fibrosis score: a noninvasive system that identifies liver fibrosis in patients with NAFLD. Hepatol Baltim Md. 2007 Apr;45(4):846–54.

13. Yip TCF, Ma AJ, Wong VWS, Tse YK, Chan HLY, Yuen PC, et al. Laboratory parameter-based machine learning model for excluding non-alcoholic fatty liver disease (NAFLD) in the general population. Aliment Pharmacol Ther. 2017 Aug;46(4):447–56.

14. Kotronen A, Peltonen M, Hakkarainen A, Sevastianova K, Bergholm R, Johansson LM, et al. Prediction of non-alcoholic fatty liver disease and liver fat using metabolic and genetic factors. Gastroenterology. 2009 Sep;137(3):865–72.

15. Ratziu V, Giral P, Charlotte F, Bruckert E, Thibault V, Theodorou I, et al. Liver fibrosis in overweight patients. Gastroenterology. 2000 Jun;118(6):1117–23.

16. Koneru K, Bhatt V, Kakrani A, Edara M, Reddy VT, Jawade PG. A study of non-alcoholic fatty liver disease-liver fat score in overweight and obese individuals. J Family Med Prim Care. 2022;11(8):4368-4374. doi:10.4103/jfmpc.jfmpc_58_22

17. Ampuero J, Pais R, Aller R, et al. Development and Validation of Hepamet Fibrosis Scoring System-A Simple, Noninvasive Test to Identify Patients With Nonalcoholic Fatty Liver Disease With Advanced Fibrosis. *Clin Gastroenterol Hepatol*. 2020;18(1):216-225.e5. doi:10.1016/j.cgh.2019.05.051

18. Lee JH, Kim D, Kim HJ, Lee CH, Yang JI, Kim W, et al. Hepatic steatosis index: a simple screening tool reflecting nonalcoholic fatty liver disease. Dig Liver Dis Off J Ital Soc Gastroenterol Ital Assoc Study Liver. 2010 Jul;42(7):503–8.
