# Supplementary material for: Fatty Liver Index (FLI) is the best score to predict MASLD with 50% lower cut-off value in women than in men
Source: Biol Sex Differ. 2024 May 17;15:43. doi: 10.1186/s13293-024-00617-z (PMC11100212; doi:10.1186/s13293-024-00617-z)
Supplement: Supplementary file 2 — Supplementary Material 2. [file 13293_2024_617_MOESM2_ESM.docx]

**Supplementary table 2. Criteria for MASLD diagnosis (1).**

| Hepatic steatosis, detected on US-imaging or histology, and at least one of the following criteria: | **BMI** | ≥25 Kg/sqm |
| --- | --- | --- |
|  | **Waist Circumference** | ≥80 cm in females, ≥94 cm in males |
|  | **HDL cholesterol** | <50 mg/dL in females, <40 mg/dL in males* |
|  | **Triglycerides** | ≥150 mg/dL * |
|  | **Hyperglycaemia** | FPG≥100 and/or HbA1c ≥5.7% * |
|  | **Hypertension** | SAP≥130mmHg and/or DAP≥85mmHg * |

*or normal values under specific pharmacological treatment.
Abbreviations: US, ultrasonography; BMI, Body Mass Index; FPG, Fasting Plasma Glucose; HbA1c, glycosylated haemoglobin; SAP, systolic arterial pressure; DAP, diastolic arterial pressure

**Reference**

1. Rinella ME, Lazarus JV, Ratziu V, Francque SM, Sanyal AJ, Kanwal F, et al. A multi-society Delphi consensus statement on new fatty liver disease nomenclature. J Hepatol. 2023 Jun 20;S0168-8278(23)00418-X.
