# Supplementary material for: Fatty Liver Index (FLI) is the best score to predict MASLD with 50% lower cut-off value in women than in men
Source: Biol Sex Differ. 2024 May 17;15:43. doi: 10.1186/s13293-024-00617-z (PMC11100212; doi:10.1186/s13293-024-00617-z)
Supplement: Supplementary file 3 — Supplementary Material 3. [file 13293_2024_617_MOESM3_ESM.docx]

**Supplementary Table 3.
Comparisons between males and females in the study population (N=1,069).**

|  | Males  (n=516) | Females  (n= 553) | *p-value* |
| --- | --- | --- | --- |
| Age (Years) | 58.3±14.2 | 57.5±15.1 | ns |
| BMI (Kg/Sqm) | 28.3±5.3 | 26.6±5.9 | <0.0001 |
| Waist Circumference (cm) | 102.6±14.2 | 94.3±15.5 | <0.0001 |
| FPG (mg/dL) | 104.8±28.7 | 94.7±25.3 | <0.0001 |
| HbA1c (mmol/mol) | 41.5±11.3 | 39±9.9 | 0.0005 |
| AST (U/L) | 24.9±11.8 | 23.2±9.6 | 0.0076 |
| ALT (U/L) | 33.6±20.8 | 27.5±14.6 | <0.0001 |
| GGT (U/L) | 39.5±36.8 | 27.9±28.2 | <0.0001 |
| Total Cholesterol (mg/dL) | 175.3±39.9 | 188.1±39.2 | <0.0001 |
| HDL Cholesterol (mg/dL) | 48.9±13.4 | 60.5±15.6 | <0.0001 |
| LDL Cholesterol (mg/dL) | 102.8±36.0 | 108.3±33.5 | 0.0104 |
| Triglycerides (mg/dL) | 130.2±73.4 | 106.5±61.5 | <0.0001 |
| Overweight (BMI≥25 and <30) | 232 (45%) | 149 (27%) | <0.0001 |
| Obesity (BMI≥30) | 148 (29%) | 144 (26%) | <0.0001 |
| Visceral Obesity | 377 (74%) | 452 (83%) | 0.0002 |
| Metabolic Syndrome | 237 (46%) | 185 (33%) | <0.0001 |
| Type 2 Diabetes | 234 (45%) | 163 (29%) | <0.0001 |
| Liver Steatosis Us Diagnosis | 370 (72%) | 278 (50%) | <0.0001 |
| Mild | 184 (36%) | 158 (29%) |  |
| Moderate | 109 (21%) | 90 (16%) |  |
| Severe | 77 (15%) | 30 (5%) |  |
| MASLD | 355 (69%) | 275 (50%) | <0.0001 |

Data are reported as mean±SD for continuous variables and as count and percentage for categorical variables. Comparisons were performed by Mann-Whitney test. Statistical significance was assessed for p-values (p) <0.05.
